# MMP proteolytic activity regulates cancer invasiveness by modulating integrins

**DOI:** 10.1038/s41598-017-14340-w

**Published:** 2017-10-27

**Authors:** Alakesh Das, Melissa Monteiro, Amlan Barai, Sandeep Kumar, Shamik Sen

**Affiliations:** Department of Biosciences & Bioengineering, IIT Bombay, Mumbai, 400 076 India

## Abstract

Cancer invasion through dense extracellular matrices (ECMs) is mediated by matrix metalloproteinases (MMPs) which degrade the ECM thereby creating paths for migration. However, how this degradation influences the phenotype of cancer cells is not fully clear. Here we address this question by probing the function of MMPs in regulating biophysical properties of cancer cells relevant to invasion. We show that MMP catalytic activity regulates cell spreading, motility, contractility and cortical stiffness by stabilizing integrins at the membrane and activating focal adhesion kinase. Interestingly, cell rounding and cell softening on stiff gels induced by MMP inhibition is attenuated on MMP pre-conditioned surfaces. Together, our results suggest that MMP catalytic activity regulates invasiveness of cancer cells by modulating integrins.

## Introduction

The mechanical properties of the extracellular matrix (ECM), especially stiffness, have been shown to regulate a gamut of cellular processes including cell proliferation, migration and differentiation^1,2^. In addition, disease states are often associated with increase in ECM stiffness, as reported in multiple cancers^3^. In breast cancer, increased deposition of collagen I and its crosslinking induces a nearly 10-fold stiffening of the mammary stroma^4^. Increase in ECM stiffness is associated with formation of stable adhesions, increased cell spreading and motility, increase in generation of cell-substrate traction forces, and increase in cell stiffness^5^. Cancer invasion through these dense matrices is associated with matrix-metalloproteinase (MMP)-mediated ECM degradation generating paths for migration^6–8^. Seminal work by Weaver and co-workers has shown that increase in ECM stiffness causes increased invadopodia-mediated ECM degradation, thereby establishing a link between increased ECM density and cancer invasiveness^9^.

In addition to ECM degradation, MMPs play diverse roles in regulating cell behavior. For example, it has been shown that outside-in signaling mediated by membrane anchored MT1-MMP is critical for regulation of the fate of skeletal stem cells^10^. The transmembrane/cytoplasmic domain of MT1-MMP has been also shown to interact with integrin β1 and regulate mammary morphogenesis via the MAPK pathway^11^. Remarkably, lack of MT1-MMP catalytic activity induced cytoskeletal and nuclear defects in fibroblasts and caused cellular senescence^12^. In melanoma cells, MMP 9 was shown to bind to CD44 and drive protease-independent migration through modulation of cell contractility^13^. MMPs have also been implicated in regulating matrix contraction by fibroblasts and keratinocyte migration during wound healing^14,15^. Together, these results highlight the diverse functions of MMPs in regulating cell behavior. However, outside of ECM degradation, the extent to which MMPs regulate cell biophysical properties relevant to invasion, remains incompletely understood.

In this study, we have probed the role of MMP catalytic activity in regulating ECM stiffness-dependent mechanoadaptation responses. Using less invasive MCF-7 cells, and highly invasive MDA-MB-231 and HT-1080 cells, we illustrate the role of MMP catalytic activity in regulating cell mechanics in the invasive cancer cells. We first show ECM stiffness modulates MMP activity in MDA-MB-231 and HT-1080 cells, but not in MCF-7 cells. Inhibition of MMP activity in the invasive cells by the broad spectrum MMP inhibitor GM6001 leads to loss of cell spreading and migration, suppression of traction forces, and cortical softening. These effects are induced by altered localization and expression of integrins, and decrease in phosphorylated focal adhesion kinase (FAK). Re-establishment of normal cell spreading on MMP-pre-conditioned substrates even in the presence of GM6001 illustrates the role of MMP catalytic activity in mediating ECM stiffness-dependent responses in highly invasive cancer cells via modulation of integrins.

## Materials and Methods

### Cell culture

MCF-7, MDA-MB-231 and HT-1080 cancer cell lines were obtained from National Center for Cell Science (NCCS) (Pune, India) and cultured in high glucose Dulbecco’s Modified Eagle Medium DMEM (Invitrogen, Cat # 11965084) containing 10% fetal bovine serum (FBS, Hi-media, Cat # RM9952) and maintained at 37 °C at 5% CO_2_ humidified atmosphere. Cells were maintained in 60 cm^2^ culture dishes (Tarsons) and passaged when 80–90% confluent using 0.25% trypsin-EDTA (Hi-media, Cat # TCL099). For culturing MCF-7 breast cancer cells, human recombinant insulin (Hi-Media, Cat # TC190) was added to the medium at a concentration of 0.01 mg/ml. For experiments, cells were first synchronized in serum free media for 18–20 hrs. prior to seeding. Further, all experiments were performed at 2% FBS concentration.

### Polyacrylamide gel (PA) preparation and ECM coating

Studies were performed with polyacrylamide gels (PA) of increasing stiffness. Gels were polymerized on circular glass coverslips of either 12 mm, 18 mm or 22 mm (Blue-star), as described elsewhere^16^. For functionalization, Sulfo-SANPAH (Thermo-scientific, Cat # 22589) at a concentration of 0.1 mM in 50 mM HEPES buffer (SRL chemicals, Cat # 63732) was added onto the surface of PA gels for 30 min under UV light at 360 nm. Gels were washed 3 times with 50 mM HEPES, and then collagen type I from rat tail (Sigma, Cat # C3867) dissolved in 1x phosphate buffer saline (PBS) was added at a concentration of 1 µg/cm^2^ overnight at 4 °C to obtain uniform surface coating.

### Cell spreading and 2D motility experiments

For stiffness dependent cell responses, cells were cultured on PA gels at a seeding density of 2 × 10^3^ cells/cm^2^ for 12-15 hrs. For cell spreading measurements, cells were fixed with 4% paraformaldehyde (PFA) (Sigma, Cat # 158127) and then stained for F-actin and nucleus using fluorescently labeled phalloidin (Invitrogen, Cat #s A-12379, A-34055) and DAPI (4′, 6-diamidino-2-phenylindole, Sigma, Cat # D9542). Fluorescent images of F-actin and nucleus were acquired using inverted microscope (Olympus IX71) at 20x magnification. Quantification of spreading analysis was done using Fiji-Image J software. Briefly, after background subtraction, images were thresholded and then cell spread area were obtained using the ImageJ-Analyze Particle tool. For probing the effect of MMPs on cell spreading, cells were allowed to adhere on to the gels for 20 mins, after which GM6001 (Abcam, Cat # ab120845) was added at a concentration of 10 μM for 12–15 hrs. Untreated cells and DMSO (MP biomedical, Cat # 02196055) treated cells served as controls. For integrin blocking experiments along with cells, RGD peptide (Sigma, Cat # G4391) at 0.2 mg/ml conc. was added in the media and incubated for 12–15 hrs. For 2D motility experiments, motility videos were acquired after 8 hrs of incubation. Time lapse imaging was performed for every 10 mins for 3 hrs duration using a temperature and CO_2_ control stage (Nikon Eclipse Ti, 20x objective). Cell speed was measured using the manual tracking plugin in Fiji-ImageJ. Directionality ratio was measured from the individual cell trajectories as described elsewhere^17^.

### Immunocytochemistry (ICC)

For immunostaining, cells were fixed after 12 hrs of culture using 4% PFA in 1x PBS for 20–25 mins, washed 3 times with 1x PBS solution to removes traces of paraformaldehyde, and permeabilized with 0.1% Triton-X 100 (Sigma, Cat # 93443) in 1x PBS solution for 3 mins. Cells were blocked with 2% BSA (Sigma, Cat # A2058) for 45 mins at room temperature (RT) before being incubated with one of the following primary antibodies overnight at 4 °C: anti-vinculin mouse monoclonal antibody (Abcam, Cat # ab18058), anti-integrin β1 rabbit polyclonal antibody (Abcam, Cat # ab155145) and anti-integrin β3 rabbit polyclonal antibody (Abcam, Cat # ab75872). The following day, cells were washed 3 times with 1x PBS and then incubated with one or more of the following secondary antibodies at room temperature (RT) for minimum 1–2 hrs: Alexa-fluor 488 anti-mouse IgG (Invitrogen, Cat # A-32723), Alexa-fluor 555 anti-rabbit IgG (Invitrogen, Cat # A-21422). Nuclei were stained with DAPI for 15 mins at RT. Finally, after washing, cells were mounted on glass-slides using Eukitt quick-hardening mounting medium (Sigma, Cat # 03989). Cells were imaged at 63x magnification using Scanning Probe Confocal Microscope (Zeiss, LSM 780) for probing integrin β1 and integrin β3 localization at cell membrane and for visualizing vinculin-stained focal adhesions. Quantification of number and size of focal adhesions (FAs) is detailed in Supplementary Method Section.

### Western blotting

For westerns, cells were lysed using RIPA buffer (Sigma, Cat # R0278) mixed with cocktail of protease and phosphatase inhibitors (Sigma, Cat # MSSAFE). Protein concentration of cell lysates were determined using Bradford assay. After loading equal amount of protein per lane, SDS-PAGE was performed. The proteins were transferred onto 0.22 μm nitrocellulose membranes (PALL Life Sciences, Cat # 66485) under ice-cold condition. Following transfer, the membranes were blocked using either 10% BSA (Sigma, Cat # A2058) or 5% skimmed milk powder (Hi-media, Cat # GRM1254) in 1x TBST (Tris-Buffered Saline and Tween-20) for 1 hr at RT, and incubated with the following primary antibodies overnight at 4 °C under mild shaking condition: anti-integrin β1 rabbit monoclonal antibody (Abcam Cat # ab155145), anti-integrin β3 rabbit polyclonal antibody (Abcam, Cat # ab75872), anti-phospho-FAK^Y397^ rabbit monoclonal antibody (Sigma, Cat # F7926), anti-β-tubulin rabbit polyclonal antibody (Abcam, Cat # ab6046), anti-β-actin mouse monoclonal antibody (Abcam, Cat # ab8226). After washing three time with 1x TBST, membranes were incubated with one of the following secondary antibodies at RT for 1 hr: HRP-conjugated anti-Rabbit IgG (Abcam, Cat # ab6721) and HRP-conjugated Anti-Mouse IgG (Abcam, Cat # ab6789). Subsequently, after washing 3 times with 1x TBST, blots were developed on X-ray films (Kodak) using a chemiluminiscent ECL kit (Pierce, Cat # 32106). Quantification of immunoblots were performed using the densitometric tool of Fiji-ImageJ software.

### Gelatin zymography

For zymography experiments, cells were cultured on soft and stiff PA gels at a seeding density of 3 × 10^3^ cells/cm^2^. Conditioned media (CM) was collected after 30 hrs of culture, and concentrated using a protein concentrator (PALL Life Sciences, Cat # MAP010C37) of 10 kDa cut-off. Zymography was performed using 8% SDS-PAGE co-polymerized with 1.5 mg/ml gelatin (Sigma, Cat # G2500). For experiments, equal amount of protein was loaded per condition and the gels were run under ice cold condition. After running, the gels were incubated with 1x renaturation buffer (2.5% Triton X-100 in distilled H_2_O) for 45 min, and equilibrated with 1x developing buffer (50 mM Tris-base, 50 mM Tris-HCL, 0.2 mM NaCl, 5 mM CaCl_2_, distilled H_2_O and pH adjusted to 7.8–8) for 45 min at RT. Next, gels were dipped in 1x developing buffer, incubated inside water bath maintained at 37 °C for minimum 20 hrs, and then stained with Coomassie Brilliant Blue (0.5% in H_2_O) till clear bands (representing protease activity) were observed. Densitometric quantification of secreted MMP levels was performed using Fiji-ImageJ software.

### Atomic Force Microscopy (AFM)

For measuring stiffness of cells and gels, pyramidal AFM probes of nominal spring constant 30 pN/nm (10 kHz, Cat # TR400PB, Olympus) were used. Exact values of cantilever stiffness were obtained using thermal calibration method. For obtaining estimates of PA gel stiffness, indentation curves were fitted till 2000 nm using Hertz model for pyramidal probe:

$${\boldsymbol{F}}{\boldsymbol{=}}\frac{{\bf{3}}}{{\bf{4}}}{\boldsymbol{\times }}{\bf{E}}\,\frac{{\bf{\tan }}({\boldsymbol{\alpha }}){{\boldsymbol{\delta }}}^{{\bf{2}}}}{({\bf{1}}{\boldsymbol{-}}{{\boldsymbol{\vartheta }}}^{{\bf{2}}})}$$

where, ***F*** represents the indentation force, ***E*** is the elastic modulus of the material, ***α*** is tip half angle, ***δ*** is indentation depth and ***υ*** is Poisson ratio of the elastic material (assumed to be 0.45 for cells and 0.5 for gels)^18^. For stiffness measurements, cells were cultured on stiff PA gels in the absence (i.e., DMSO only) and presence of GM6001 for 12–15 hrs duration. For measuring cell cortical stiffness, cells were probed slightly off their center to get the best estimate of cortical stiffness, and the first 500 nm of indentation data was fit using the above Hertz model.

### Adhesion experiments

For AFM based RGD-integrin binding measurements, cells were cultured on stiff PA gels in the absence (i.e., DMSO only) and presence of GM6001 for 12–15 hrs duration. Spherical probes (67 kHz, Novascan), 4.5 μm in diameter were coated with 10 μg/ml solution of poly-L-lysine (Sigma, Cat # P4707) for 20 mins and then treated with 0.5% glutaraldehyde (Sigma, Cat # G7651) for 20 mins^19^. Next, probes were dipped in 0.1 mg/ml solution of RGD peptide (Sigma, Cat # G4391) for 30 mins, washed 3–4 times in distilled H_2_O, and then dried in a vaccum dessicator for 30 mins. After tip calibration using thermal noise method, RGD-integrin binding studies were performed. While indenting, probe was held at the cell surface for 10 secs to allow formation of integrin-RGD bonds, and then retracted at a tip speed of 3–4 μm/sec. Analysis of maximum adhesion force was performed using Igor Pro 6.22 A software.

### Traction force Microscopy (TFM)

For TFM studies, cells were seeded sparsely (1000 cells/cm^2^) on collagen coated stiff PA gels co-polymerized with 1 μm fluorescent beads (Sigma, Cat # L9654), and cultured in the absence (i.e., DMSO only) and presence of GM6001 for 12–15 hrs duration. Images of beads were acquired at 20x magnification (using a Nikon Eclipse Ti inverted microscope) before and after bursting cells using 0.1% Triton X-100 in H_2_O. Data analysis was done using custom written codes in MATLAB 7.8 as described elsewhere^20^.

### Preparation and characterization of 3D collagen gels

For collagen gel preparation, high protein collagen type I solution (Corning, Cat # 354236) was mixed with 10x PBS kept at 4 °C. Next, ice cold plain DMEM was added and pH was adjusted to 7.2–7.4 using 1 N NaOH solution. After adjusting pH, collagen gel mixture was kept on ice for 10 mins, then 2 × 10^3^ cells were mixed with collagen gel mixture and immediately kept inside CO_2_ incubator for 1 hr. to allow gel formation. After that 10% DMEM was added and allowed the cell to acclimatize for minimum 4 hrs inside CO_2_ incubator. Then, time lapse movies of untreated, DMSO treated, GM6001 treated (10 µM) cells inside collagen gel were captured for 12 hrs at 20 mins interval using temperature and CO_2_ controlled stage (Nikon Eclipse Ti, 10x objective). Cell speed and directionality ratio was obtained as mentioned above. For characterization, collagen hydrogels were snap frozen with liquid N_2_ mounted on the Cryo-unit (PP3000T, Quorum) and fractured with a blade. The frozen samples were then sputter coated with a thin layer of platinum, and Cryo-SEM images obtained with a JSM-7600F FEG-SEM with an acceleration voltage of 5 kV. Pore area of hydrogels was quantified by using Analyse-particle tool of Fiji-ImageJ software.

### Statistics

For statistical analysis, data was first tested for normality using Kolmogorov-Smirnov normality test in Origin 9.1. Based on the outcome of the normality tests, either parametric or non-parametric statistical test were performed. For parametric data, one-way ANOVA/two-way ANOVA was performed to assess statistical significance, and Fisher post-hoc test was used to compare the means. For non-parametric data, Kruskal-Wallis ANOVA/Mann-Whitney test was performed. All statistical analysis was performed using Origin 9.1 with *p* value < 0.05 considered to be statistical significant.

## Results

### MMP activity is essential for eliciting ECM stiffness-dependent responses in invasive cancer cells

**Figure 1:**
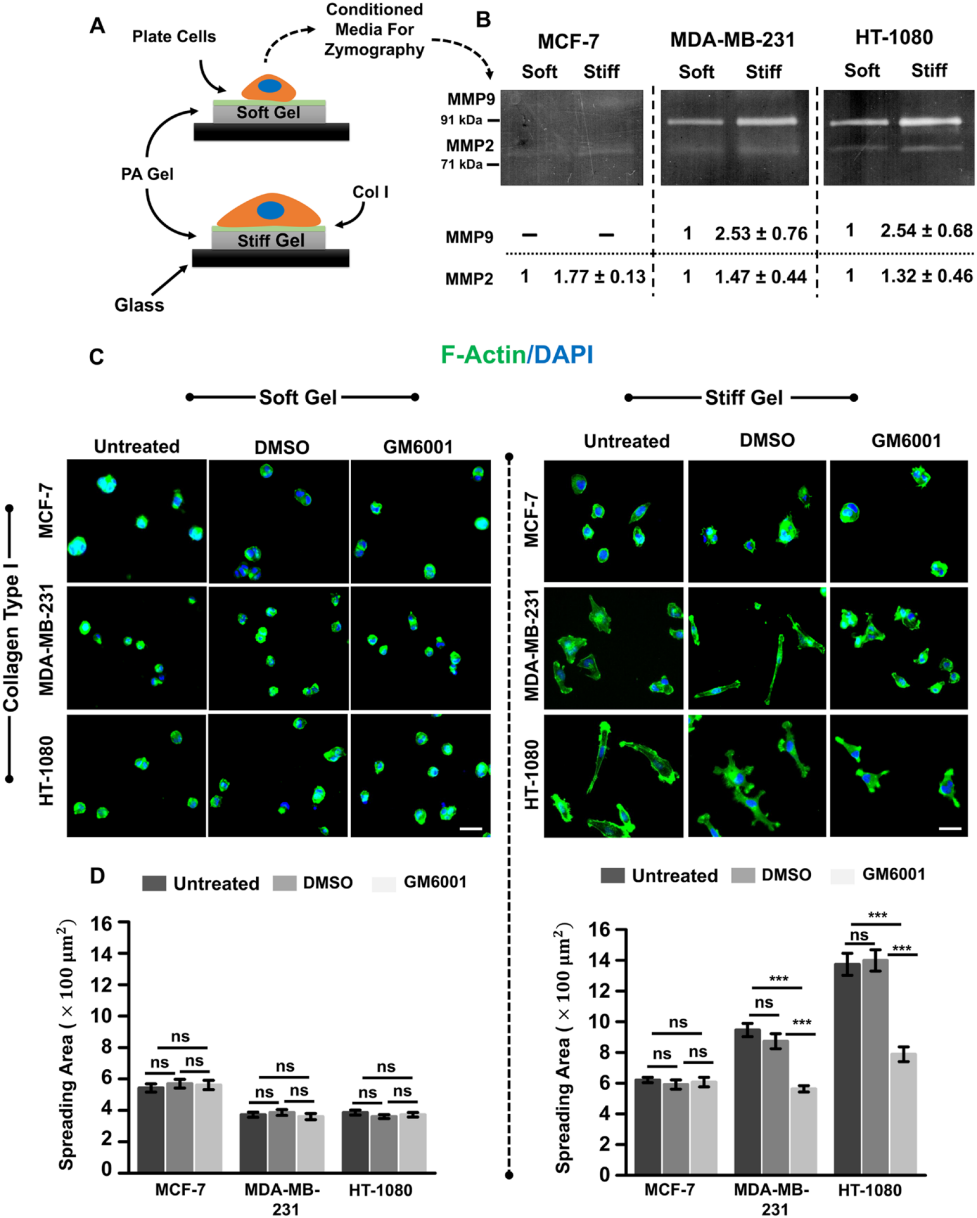
Influence of MMP inhibition on stiffness-dependent cell spreading. (**A**) MCF-7, MDA-MB-231 and HT-1080 cells were cultured sparsely on collagen type I-coated soft and stiff polyacrylamide (PA) hydrogels for desired time duration. (**B**) Assessment of stiffness-dependent activity of matrix metalloproteinases (MMPs) (MMP 2, and 9) using gelatin zymography. Conditioned medium was collected after 30 hrs of incubation. Densitometric quantification of secretion levels of soluble MMPs in conditioned media by MCF-7, MDA-MB-231 and HT-1080 cells seeded on soft and stiff substrates reveals increase in MMP activity on stiffer gels (*n* = 2). (**C**) Representative F-actin (green) and DAPI (blue) stained images of untreated, DMSO treated and GM6001 treated MCF-7, MDA-MB-231, and HT-1080 cells seeded on soft and stiff gels. Scale bar = 50 μm. (**D**) Quantitative analysis of cell spreading of untreated, DMSO treated and GM6001 (GM) treated MCF-7, MDA-MB-231 and HT-1080 cancer cells seeded on soft and stiff gels (*n* = 2–3, at least 90–140 cells per condition). Stars denote statistical significance (***p < 0.001, **ns**: not significant). Statistical significance was determined using Kruskal-Wallis ANOVA/Mann-Whitney test. Error bars represent standard error of mean (±SEM).

Most epithelial cancers are associated with increase in ECM stiffness driven by increased deposition and crosslinking of collagen I^21^. Since increase in ECM stiffness has been associated with increased invasiveness^22^, we hypothesized that ECM stiffness positively regulates MMP activity. To test this, experiments were performed on collagen-coated soft (0.46 kPa) and stiff (4.6 kPa) PA gels (Supp. Figure 1A,B) using the less invasive MCF-7 cells (henceforth MCF7)^23^ and two highly metastatic cell lines (MDA-MB-231 and HT1080 (henceforth MDAMB and HT, respectively) (Fig. 1A). MMP activity on these gels was assessed using gelatin zymography using conditioned media collected from cells cultured on the gels for 30 hrs. Zymography revealed stiffness-dependent increase in MMP 2 and MMP 9 activity in MDA and HT cells; in contrast, in MCF7 cells, very low MMP activity was detected (Fig. 1B).

To next probe the influence of stiffness-dependent increase in MMP activity on stiffness-dependent cell spreading, experiments were performed with untreated cells, vehicle treated cells (DMSO), and cells cultured in the presence of the broad spectrum MMP inhibitor GM6001 (GM). Cell spreading was assessed after culturing on PA gels for 12–15 hrs. On soft gels, all the three cell types cells exhibited rounded morphology consistent with previous studies^24^, with GM treatment having no effect (Fig. 1C,D). While cell spreading increased on the stiff gels, GM treatment significantly suppressed cell spreading of invasive MDAMB and HT cells, with ~40% drop in spreading of MDAMB cells and ~50% drop in spreading of HT cells. In contrast, there was no significant change in MCF7 cell spreading. Collectively, these results suggest that ECM stiffness-induced increase in MMP activity is essential for cell spreading of MDAMB and HT cells on stiff gels.

### MMP activity regulates cell adhesion and motility

**Figure 2:**
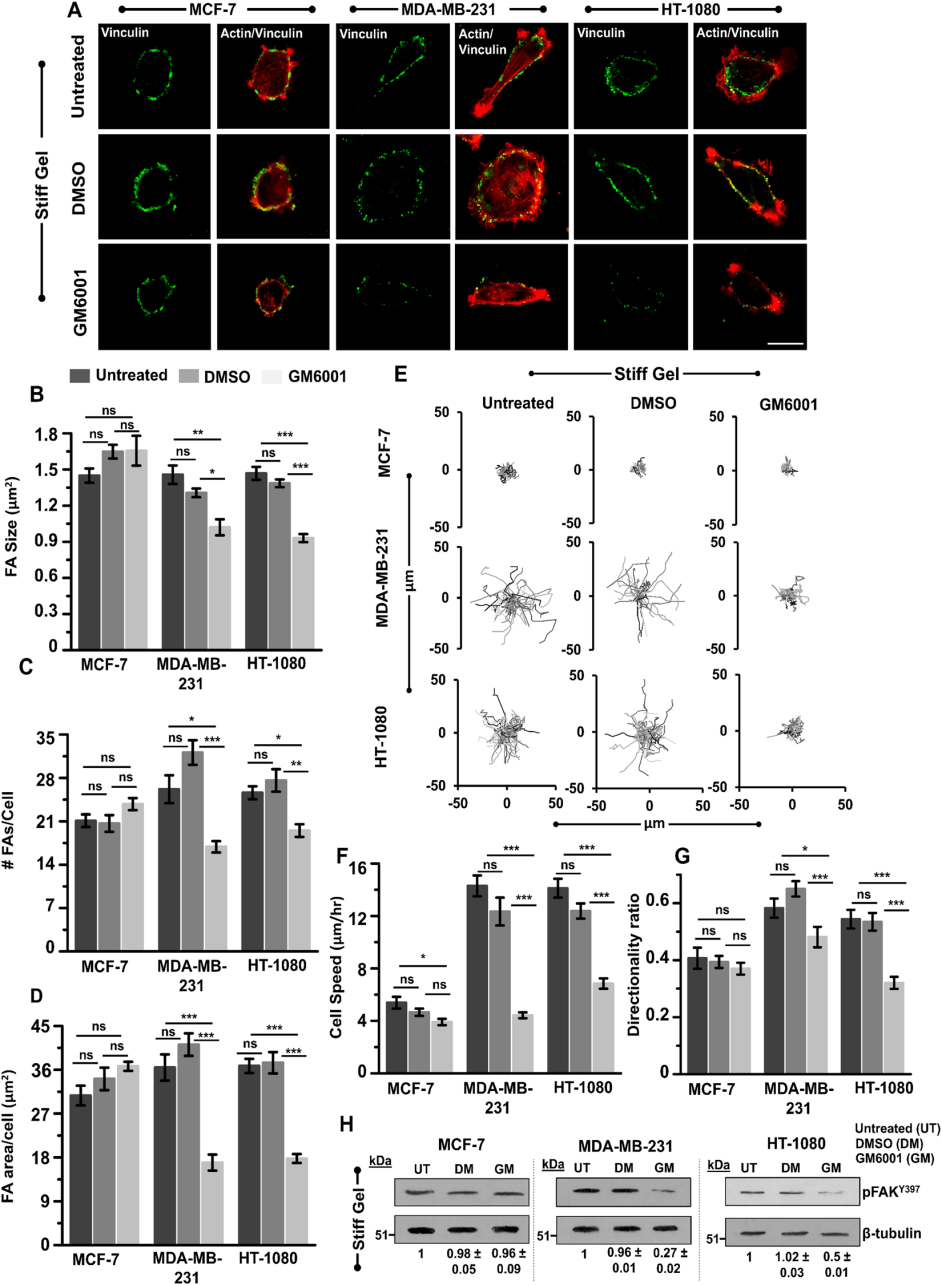
Influence of MMP inhibition on focal adhesion and cell motility of MCF-7, MDA-MB-231 and HT-1080 cells. (**A**) Immunostained images of the focal adhesion protein vinculin (green) and F-actin (red) in untreated, DMSO treated and GM treated cells cultured on stiff PA gels. Scale bar = 20 μm. (**B**,**C**,**D**) Quantitative analysis of size, number of focal adhesion and focal adhesion area per cell in untreated, DMSO treated and GM treated cells (*n* = 2, at least 30–35 cells per condition; **p < 0.01, *p < 0.05, **ns**: not significant). Statistical significance was determined by one-way ANOVA/Fisher test. Error bars represent standard error of mean (±SEM). (**E**) Representative random cell migration trajectories of untreated, DMSO treated and GM treated cells cultured on stiff PA gels. (**F**,**G**) Quantitative analysis of cell speed (*n* = 2, 45–50 cells per condition; ***p < 0.001, **ns**: not significant) and directionality ratio (*n* = 2, 35–45 cells per condition; ***p < 0.001; *p < 0.05; **ns**: not significant) of untreated, DMSO treated and GM treated cells cultured on stiff PA gels. Statistical significance was determined by one-way ANOVA/Fisher test. Error bars represent standard error of mean (±SEM). (**H**) Western blot analysis of phosphorylated-focal adhesion kinase (pFAK^Y397^) in untreated, DMSO treated and GM treated cells cultured on stiff PA gels. β-tubulin served as a loading control (*n* = 2).

To probe the mechanism by which MMP inhibition leads to reduction in cell spreading, cells on stiff gels were stained for the focal adhesion protein (FA) vinculin (Fig. 2A). Quantitative analysis of the number and size of focal adhesions (method of quantification detailed in Supp. Methods and Supp. Figure 2) revealed that GM treatment led to reduction in both size and number of FAs in MDAMB and HT cells, but not in MCF7 cells (Fig. 2B,C). Taking the product of the average FA number and the average FA size allowed us to compare the total focal adhesion area in untreated, DMSO treated and GM treated cells. Quantification revealed ~40–50% drop in in GM treated cells (Fig. 2D), which was comparable to the drop in cell spreading (Fig. 1C,D).

Next, to determine if GM induced loss of focal adhesions led to motility defects, random cell motility experiments were performed on stiff gels with untreated, DMSO treated, and GM treated cells. For these experiments, untreated, DMSO treated and GM treated cells were allowed to spread for 8 hrs, and then cell motility experiments were performed for 3 hrs (Supp. Videos V1–V6). As seen from the representative cell trajectories, GM treatment had negligible effect on the motility of MCF7 cells (Fig. 2E). However, in both MDAMB and HT cells, the lengths of the traces of GM treated cells were significantly smaller than those of untreated cells (Fig. 2E); quantification of cell trajectories revealed significant suppression in cell speed (Fig. 2F) and directionality ratio (Fig. 2G) in GM treated invasive cells.

Next, to probe the importance of MMP activity is mediating 3D cell invasion, we performed 3D invasion assay by using collagen 3D gels (Supp. Figure 3). Specifically, cells were encapsulated in 1 mg/ml collagen gels with average pore area of ~4 μm^2^ (Supp. Figure 3A,B). Since nuclear translocation is rate limiting for migration under confinement^25^, and the pore size of these gels (~2–4 μm in diameter, obtained by approximating the pore as a circle) was smaller than nuclear width of all the three cell types (Supp. Figure 3C,D), this gel concentration was enough to inhibit cell migration by providing steric hindrance. Cell invasiveness was measured by tracking motility of untreated, DMSO treated and GM treated cells for 12 hrs duration. Similar to our 2D results, both MDAMB and HT cells exhibited faster motility than MCF7 cells in 3D collagen gels, with GM treatment inhibiting cell motility and persistence of MDAMB and HT cells (Supp. Figure 3E–G). Our results are consistent with findings by others groups who have shown that MMP mediated ECM degradation is critical for 3D invasion through matrices with sub-nuclear sized pores^26–28^.

The combination of loss of focal adhesions, reduction in motility in 2D, and reduction in invasion in 3D induced by GM treatment suggests that MMP activity is critical for cell spreading, migration and invasion. To probe if these alterations can be correlated with alterations in the phosphorylation levels of focal adhesion kinase (FAK)—a key signaling molecule involved in cell adhesion and migration^29–31^, levels of phosphorylated FAK (pFAK^Y397^) were assessed in MDAMB, HT and MCF7 cells. Densitometric analysis of immunoblots revealed a significant drop in the expression level of pFAK^Y397^ in GM treated MDAMB and HT cells, but not in MCF7 cells (Fig. 2H).

Collectively, these results indicate that MMP activity regulates spreading and motility of MDAMB and HT cells on stiff gels at least in part via formation of focal adhesions and FAK^Y397^ phosphorylation.

### Inhibition of MMP activity suppresses cell-ECM tractions and induces cell softening in invasive cancer cells

**Figure 3:**
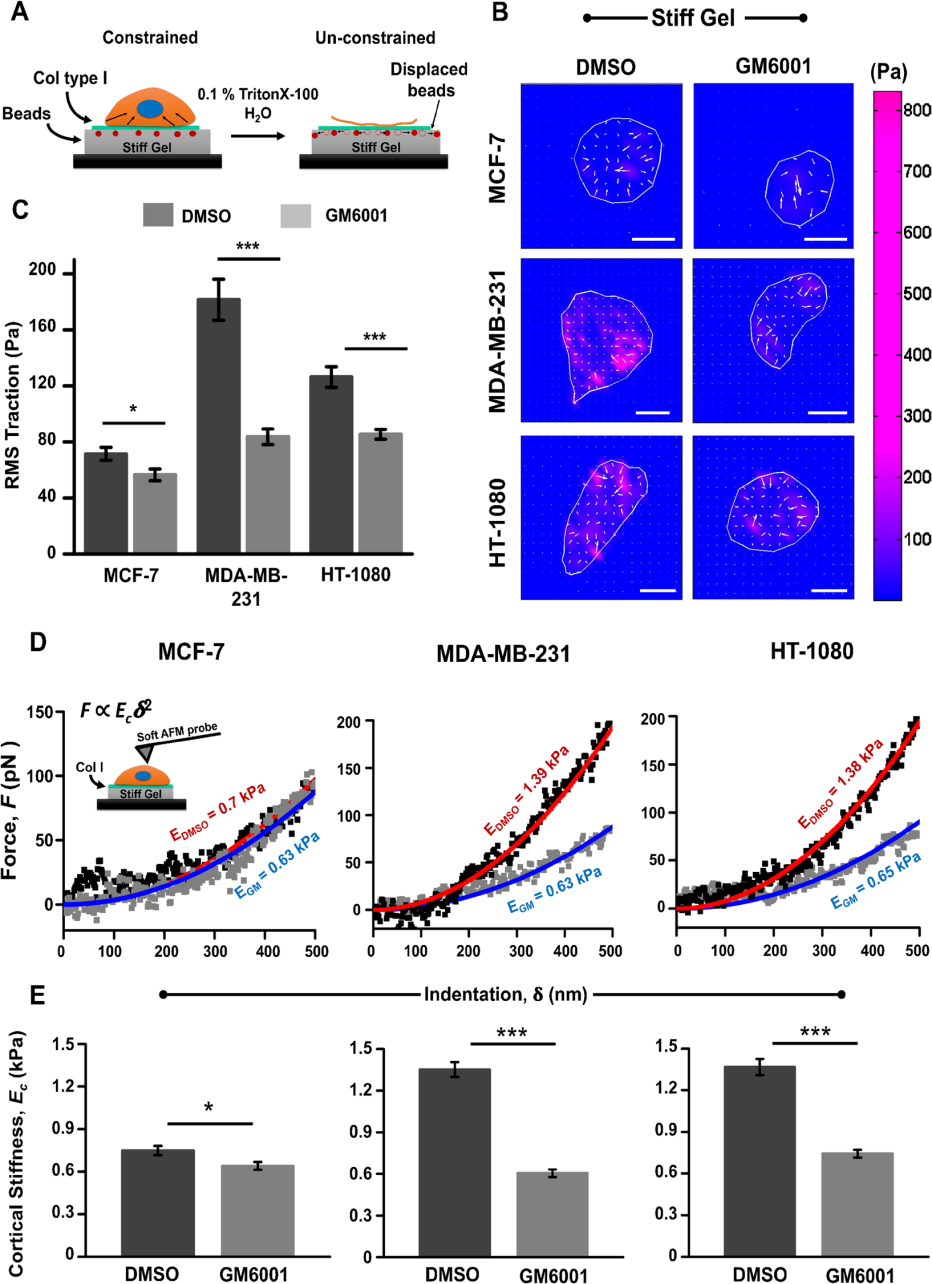
Influence of MMP inhibition on cell-ECM tractions and cortical stiffness of MCF-7, MDA-MB-231 and HT-1080 cells. (**A**) Schematic of traction force microscopy (TFM). Forces exerted by the cells are calculated based on deformations of beads embedded in the gel. The ‘constrained’ condition corresponds to the case when the cell is attached to the substrate and exerting tractions. The ‘unconstrained’ condition corresponds to the case when the cell is removed and the beads relax to their original positions. (**B**) Representative traction force maps of DMSO and GM treated cells grown on stiff PA gels (Scale bar = 20 µm). (**C**) Quantitative analysis of root mean square traction (RMS traction) of DMSO treated and GM6001 treated cells grown on stiff PA gels (*n* = 3, 35–45 cells per condition). Stars denote statistical significance (***p < 0.001, ns: not significant). Statistical significance was determined by one-way ANOVA/Fisher test. Error bars represent standard error of mean (±SEM). (**D**) Representative force-indentation curves of DMSO treated and GM treated cells. First 500 nm of the force curves were fit with Hertz equation to obtain estimates of cortical stiffness. (**E**) Quantitative analysis of cell cortical stiffness of DMSO treated and GM treated cells (*n* = 3–4, 100–140 cells per condition; ***p < 0.001, *p < 0.05). Statistical significance was determined by Mann-Whitney test. Error bars represent standard error of mean (±SEM).

Thus far, our results suggest that in highly invasive MDAMB and HT cells, MMP inhibition induces loss of focal adhesions and downregulates pFAK^Y397^. Next to probe the impact of this perturbation on cytoskeletal organization, we used traction force microscopy (TFM) (Fig. 3A) and AFM to measure cell generated tractions and cell cortical stiffness, respectively. Strikingly, GM treatment was found to inhibit cell-ECM tractions significantly in MDAMB and HT cells, but not in MCF7 cells (Fig. 3B,C). In addition, analysis of cortical stiffness of cells by AFM revealed significant cortical softening in both MDAMB and HT cells, but not in MCF7 cells (Fig. 3C,D). Taken together, these results indicate that inhibition of MMP activity in invasive cancer cells perturbs cell mechanical properties via reduction in cell-ECM traction and by inducing cell cortical softening.

### Inhibition of MMP activity perturbs integrin β1 levels and its membrane localization

**Figure 4:**
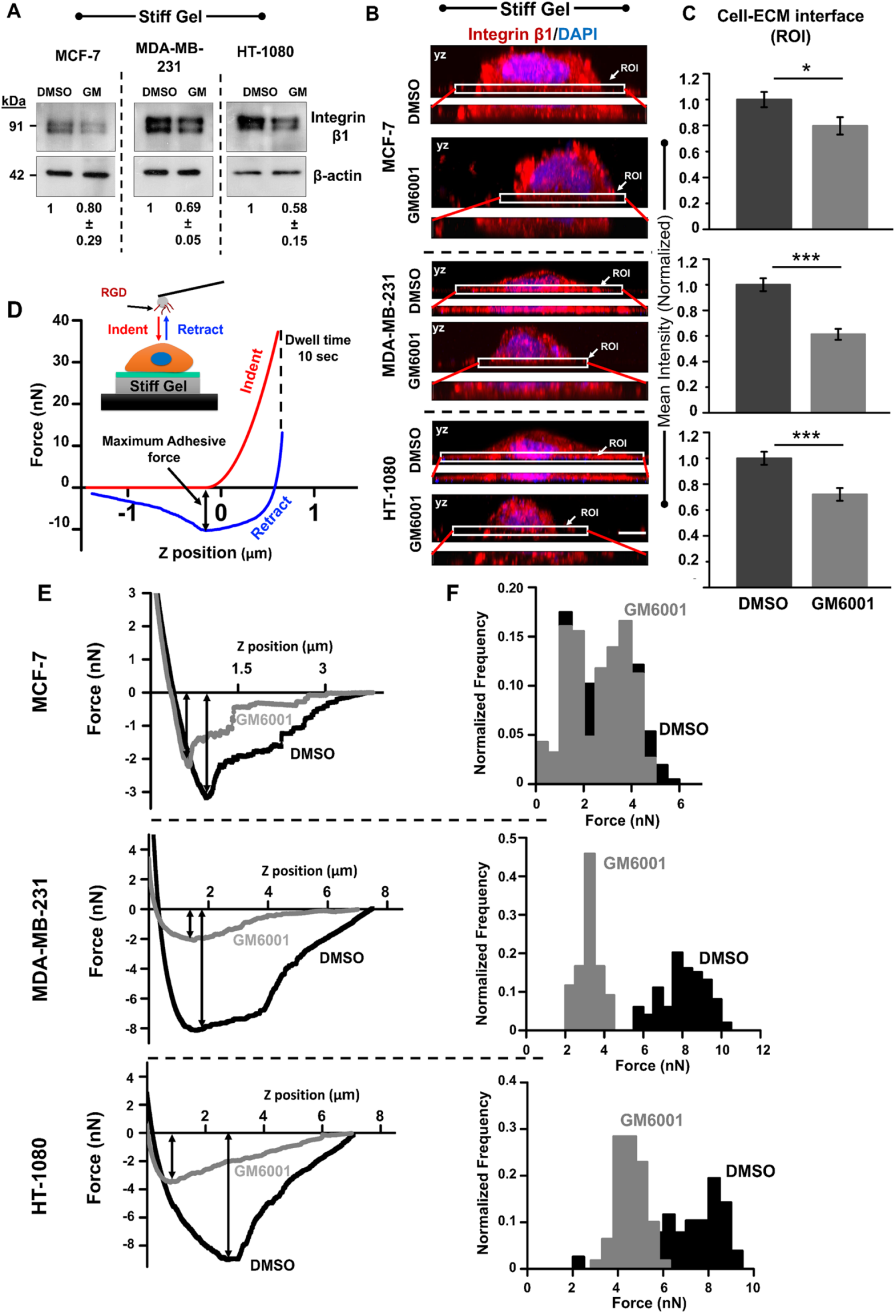
Influence of MMP inhibition on integrin expression levels and its membrane localization in MCF-7, MDA-MB-231 and HT-1080 cells. (**A**) Western blot analysis of integrin β1 in DMSO treated and GM treated cells cultured on stiff PA gels. β-actin served as a loading control (*n* = 2). (**B**) Representative maximum intensity projection images (along the height of the cells (YZ plane)) of DMSO treated and GM treated cells stained for integrin β1 (red) and DNA binding dye DAPI (blue) (Scale bar = 8 µm). Insets show localization of integrin β1 at the basal cell-ECM interface (ROI, region of interest). (**C**) Quantification of mean integrin β1 intensity at the basal surface in DMSO treated and GM treated cells (normalized with respect to mean intensity of DMSO treated cells) (*n* = 2, 25 cells per condition, ***p < 0.001, *p < 0.05). Statistical significance was determined by one-way ANOVA/Fisher test. Error bars represent standard error of mean (±SEM). (**D**) Quantification of membrane-localized integrins using Atomic Force Microscopy (AFM). Schematic shows probing of cells using 0.1 mg/ml RGD-functionalized spherical probes of diameter 4.5 µm. For this experiment, after indentation, tip was held in position for 10 secs (i.e., dwell time = 10 secs) to allow formation of integrin-RGD bonds, and then retracted. Representative force curve showing indentation of cell and breakage of integrin-RGD bonds during retraction. Maximum adhesive force corresponds to the maximum number of bonds formed. (**E**) Representative retraction curves in DMSO treated and GM treated cells cultured on stiff PA gels. (**F**) Histogram of maximum adhesive force in DMSO treated and GM treated cells cultured on stiff PA gels (*n* = 3–4, 100 cells per condition).

Since MMPs are known to bind to integrins^32^, and their association is crucial for various cellular processes including cell migration^33^, we hypothesized that inhibition of MMP activity perturbs integrins levels and/or its localization at the membrane thereby influencing cell spreading and motility. Indeed, inhibition of integrins by soluble RGD peptide^34–36^ led to reduction in cell spreading across all the three cell types, and also induced cell softening (Supp. Figure 4A–C). To probe if cell rounding observed by GM treatment can be attributed to perturbed expression and/or localization of integrin β1, levels of integrin β1were compared between DMSO treated and GM treated cells. Western blots of cell lysates collected from DMSO treated and GM treated samples revealed suppression of integrin β1 in all the cells, with maximal drop in HT cells and minimal drop in MCF7 cells (Fig. 4A). To see if integrin localization was also perturbed by GM treatment, cells were immunostained with an integrin β1 antibody that binds to the extracellular domain and 3D imaging was performed to determine the membrane localization of integrin β1. Confocal imaging revealed suppression of integrin β1 localization at the basal cell-ECM contact surface in GM treated MDAMB and HT cells (Fig. 4B). This was also quantitatively confirmed by comparing the mean fluorescence intensity in the region of interest (ROI) (i.e., basal surface) shown in the zoomed-in inset images (Fig. 4B inset). Tracking the mean intensity automatically corrected for differences in the extent of cell spreading between DMSO treated and GM treated conditions. Plotting of the mean normalized intensity, i.e., mean intensity normalized with respect to the DMSO treated condition, revealed ~20–40% drop in integrin membrane localization in GM treated cells (Fig. 4C).

To functionally probe the effect of drop in integrin levels and its altered localization at the cell membrane, an adhesion assay was performed wherein a RGD functionalized spherical AFM tip was brought in contact with a cell, adhesive bonds were allowed to form for 10 secs, after which the tip was retracted (Fig. 4D). The maximum adhesive force, i.e., the peak force observed during retraction, is indicative of the maximum number of bonds formed (Fig. 4D), and provides a way of quantifying the extent of GM-induced loss of integrin activity near cell membrane. Tracking of the maximum adhesive force thus allowed us to estimate the extent of integrin activity in DMSO treated and GM treated cells. In DMSO treated cells, maximum adhesive forces observed in MDAMB and HT cells were upto 2-fold higher than those observed in MCF7 cells (Fig. 4E,F). While no significant differences in the maximum adhesive force was observed between untreated and GM treated MCF7 cells, GM treatment led to significant decrease in the maximum adhesive force in both MDAMB and HT cells, with maximum effect observed in MDAMB cells (Fig. 4E,F). Together, these results suggest that GM induced defects in cell spreading and motility in highly invasive cells is associated with loss of integrin levels and its localization at the plasma membrane.

### MMP proteolytic activity enables mechanoadaptation via integrins

**Figure 5:**
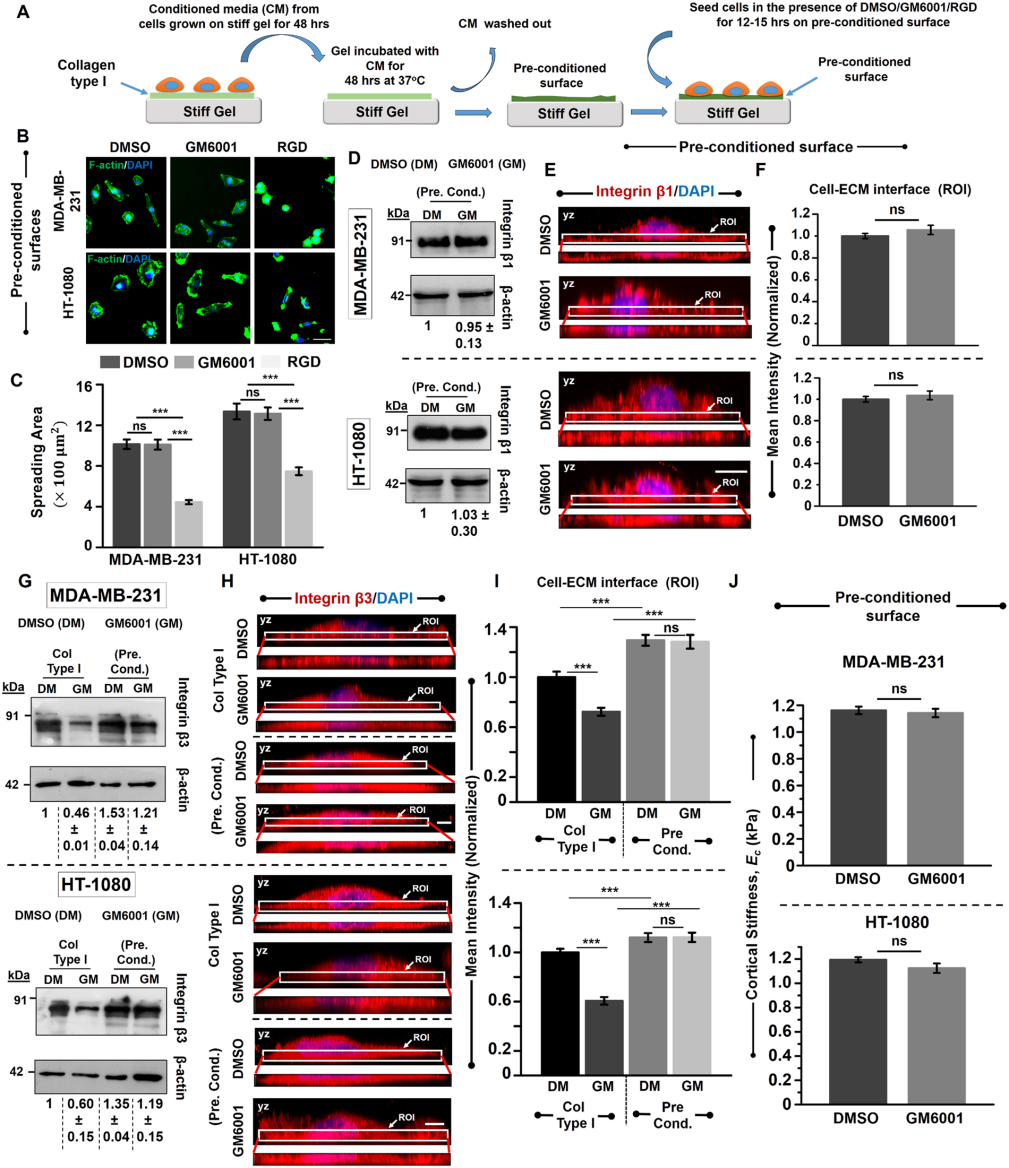
Rescue of invasive phenotype of MDA-MB-231 and HT-1080 cancer cells on MMP pre-conditioned surfaces. (**A**) Schematic of generating pre-conditioned surfaces. Conditioned media (CM) was collected from MDA-MB-231 and HT-1080 cancer cells cultured for 48 hrs on Col I coated stiff gels. Fresh Col I coated stiff gels were then incubated with the collected CM for 48 hrs at 37 °C. Subsequently, the gels were washed and a fresh batch of MDA-MB-231 and HT-1080 cells were cultured in the presence of DMSO/GM/RGD for 12–15 hrs. (**B**) Representative fluorescent images of DMSO treated, GM treated and RGD treated cells on pre-conditioned surfaces (Scale bar = 50 µm). Cells were stained for F-actin (green) and DAPI (blue) respectively. (**C**) Quantitative analysis of spreading of DMSO treated, GM treated cells and RGD treated cells (along with RGD peptide, equivalent amount of DMSO was also added) on MMP-pre-conditioned surfaces (*n* = 2, 150–170 cells per condition; ***p < 0.001, **ns**: not significant). Statistical significance was determined by Kruskal-Wallis ANOVA/Mann-Whitney test. Error bars represent standard error of mean (±SEM). **(D**) Western blot analysis of integrin β1 in DMSO treated and GM treated cells cultured on pre-conditioned surfaces. β-actin served as loading control (*n* = 2). (**E**) Representative maximum intensity projection of DMSO treated and GM treated cells (along the height of the cells (YZ plane)) cultured on pre-conditioned surfaces and stained for integrin β1 (red) and DNA binding dye DAPI (blue) (Scale bar = 10 µm). Insets show localization of integrin β1 at the basal cell-ECM interface (ROI, region of interest). (**F**) Quantification of mean integrin β1 intensity at the basal surface in DMSO treated and GM treated cells (normalized with respect to mean intensity of DMSO treated cells) (*n* = 2, 30 cells per condition; **ns**: not significant). Statistical significance was determined by one-way ANOVA/Fisher test. Error bars represent standard error of mean (±SEM). (**G**) Western blot analysis of integrin β3 in DMSO treated and GM treated cells cultured on Col I/pre-conditioned surfaces. β-actin served as loading control (*n* = 2). (**H**) Representative maximum intensity projection of DMSO treated and GM treated cells cultured on Col I/pre-conditioned surfaces and stained for integrin β3 (red) and DNA binding dye DAPI (blue) (Scale bar = 5 µm). Insets show localization of integrin β3 at the basal cell-ECM interface (ROI, region of interest). (**I**) Quantification of mean integrin β3 intensity at the basal surface in DMSO treated and GM treated cells (normalized with respect to mean intensity of DMSO treated cells cultured on Col I coated gels) (*n* = 2, 25–30 cells per condition; ***p < 0.001; **ns**: not significant). Statistical significance was determined by two-way ANOVA. Error bars represent standard error of mean (±SEM). (**J**) Quantitative analysis of cell cortical stiffness of DMSO treated and GM treated cells on pre-conditioned substrates (*n* = 2, 110–130 cells per condition; **ns**: not significant). Statistical significance was determined by Mann-Whitney test. Error bars represent standard error of mean (±SEM).

While our results clearly demonstrate that GM treatment leads to alterations in focal adhesions and cell biophysical properties, it remains unclear if the effects are purely a consequence of extracellular protease activity or can be attributed to other functions of MMPs. To address this question, conditioned media (CM) collected from cells cultured on stiff gels for 48 hrs was added onto fresh Col I coated stiff gels and incubated at 37 °C under sterile conditions (Fig. 5A). After 48 hrs, the CM was washed out and fresh cells were seeded onto the pre-conditioned surfaces for 12–15 hrs in the presence of DMSO/GM/RGD. While GM treated cells spread to the same extent as that of DMSO treated cells, RGD treatment induced cell rounding on the pre-conditioned surfaces (Fig. 5B,C) similar to RGD induced cell rounding observed on un-conditioned stiff gels (Supp. Figure S4). Consistent with similar spreading between DMSO treated and GM treated cells on pre-conditioned surfaces, integrin expression was nearly identical in DMSO treated and GM treated cells (Fig. 5D). Furthermore, basal localization of integrin β1 remained unchanged in both MDAMB and HT cells across the two conditions (Fig. 5E,F).

To further probe the mechanism by which MMP proteolytic activity influences cell adhesion and spreading, we hypothesized that ECM degradation exposes hidden RGD like motifs of collagen type I thereby allowing other integrin sub-types to engage^37,38^. Since integrin β3 can bind to RGD^39^, we probed expression levels of integrin β3 in DMSO and GM treated MDAMB and HT cells cultured on Col I-coated stiff gels as well as on pre-conditioned surfaces. While integrin β3 expression was found to be significantly lower in GM treated cells compared to DMSO controls on Col I-coated surfaces, robust spreading of GM treated cells on pre-conditioned surfaces was associated with more than 2-fold integrin β3 expression compared to that on Col I-coated gels (Fig. 5G). In line with higher integrin β3 expression observed on pre-conditioned surfaces, immunostaining revealed increased integrin β3 localization at the cell-ECM interface in both DMSO treated and GM treated cells on the pre-conditioned surfaces compared to that on Col I-coated surfaces (Fig. 5H,I).

In line with normal cell spreading and robust F-actin staining observed in GM treated cells on the pre-conditioned surfaces, probing of cell mechanical properties revealed no differences in cortical stiffness of DMSO treated and GM treated cells (Fig. 5H). Collectively, our results suggest that MMP mediated pre-conditioning of collagen type I enables cell mechanoadaptation via integrins.

## Discussion

In this work, we have demonstrated the role of MMPs in regulating stiffness-dependent responses in invasive cancer cells. Our results reveal that stiffer substrates enhance MMP activity. Interestingly, inhibition of MMP activity induces cell rounding through loss of focal adhesions, suppresses traction generation, cell motility and cell invasiveness, and softens cells. These phenotypic defects could be rescued if cells were seeded on MMP pre-conditioned surfaces. Overall, our results suggest that ECM stiffening-induced up-regulation of MMP extracellular proteolytic activity contributes to maintenance of invasive phenotype in MDAMB and HT cancer cells.

Epithelial cancers are associated with ECM stiffening^40^. Such stiffening, achieved by increased deposition and crosslinking of fibrillar ECM proteins including collagen, leads to reduction in pore size^21,41^. This should ideally limit cancer invasiveness by providing increased steric hindrance; instead, cells become more invasive^5,22,42^. Together, our 2D cell motility and 3D invasion experiments suggest that increased invasiveness is a consequence of increased MMP activity in invasive MDAMB and HT cells. The absence of stiffness-dependent changes in MMP activity observed in non-metastatic MCF7 cells suggests that stiffness-dependent modulation of MMP activity is a property of highly invasive cancer cells. Our results further suggest that MMP activity mediates stiffness adaptation by modulating integrins, with inhibition of MMP activity suppressing mechanosensing leading to defective cell spreading and motility.

Previous studies have shown that extracellular activity of MMPs generates cleaved fragments of ECM components^43^, which serve as chemotactic cues and stimulate directed migration of fibroblasts^44^ and keratinocytes during wound healing and tissue repair processes^45^. In addition, MMP mediated cleavage of laminin-5 and collagen IV have also been shown to expose cryptic binding sites which aid in cell migration^46,47^. MMP 1 mediated collagen I degradation is also crucial for epithelial cell migration during wound closure^48^. GM-induced loss of cell spreading and motility defects may be attributed to the absence of cleaved ECM fragments and/or exposure of cryptic domains, as cell spreading is rescued on MMP pre-conditioned matrices even in the presence of GM. Consistent with this, we observe increase in integrin β3 localization in MDAMB and HT cells at the basal cell surface on MMP pre-conditioned surfaces.

Several reports have documented regulation of outside-in signaling by MMPs. For example, Weiss and co-workers have shown that catalytic activity of MT1-MMP regulates skeletal stem cell fate via integrin β1 signaling^10^. Another group has shown that MMP 1 activity at the cell surface of platelets can induce thrombosis via PAR1 (protease activated receptor) signaling^49^. MMP substrate specificity is not just limited to ECM components, but also includes growth factors such as TGF-β^50^, which is known to play a significant role in cancer progression^51^. Studies suggest that MMP 9 and MMP 2 can proteolytically activate TGF-β at the cell periphery and induce angiogenesis and tumor invasion^52^. In our study, MMP inhibition-induced downregulation of pFAK^Y397^, suppression of cell-ECM tractions, softening of cell cortex and loss of integrins at the cell surface in invasive MDAMB and HT cancer cells illustrates the importance of MMP activity in regulating localization of cell surface receptors and its downstream signaling processes. However, the exact mechanism of modulation of integrin stability by MMP activity remains unknown. Recent studies have shown that co-localization and interaction between MT1-MMP and integrin β1 is essential for mammary morphogenesis, with perturbation of either one of the two suppressing morphogenesis^11^. In addition, both MMP 2 and MMP 9 have also been shown to interact with integrins^53^. Therefore, GM-induced alterations in cell phenotype are probably brought about by alterations in the interaction between integrins and MMPs.

Recent studies in fibroblasts have established a mechanistic link between MT1-MMP mediated extracellular proteolysis and cytoskeletal and nuclear organization^12^. Similarly, in skeletal stem cells, ECM remodeling has been shown to regulate RhoGTPase signaling and pericellular rigidity^10^. Previously, Sheetz and co-workers have demonstrated the role of integrin β1 in determining adhesion strength and integrin β3 in regulating mechanotransduction^54^. In our study, cell rounding on stiff surfaces in the presence of GM and phenotypic rescue of MDAMB and HT cells on MMP pre-conditioned surfaces even in the presence of GM suggests that MMP pre-conditioning exposes hidden RGD-like motifs in collagen^37,55–57^. Subsequently, exposure of these RGD-like motifs induce expression of other integrin sub-types (integrin β3 in our case)^39^ thereby regulating mechanoadaptation on stiff collagen coated surfaces. Overall, our results thus illustrate the existence of a crosstalk between MMP mediated ECM remodeling and cell mechanical properties mediated via integrins.

In summary, our results demonstrate ECM stiffness-dependent modulation of MMP activity in invasive cancer cells, and the role of this increased MMP activity in sustaining the invasive phenotype of cancer cells through modulation of integrins. Future studies will specifically focus on understanding the contributions of distinct soluble and membrane bound MMPs in regulating cell biophysical properties.

## Electronic supplementary material

Supplementary information

MDA-MB-231 Untreated

MDA-MB-231 DMSO

MDA-MB-231 GM6001

HT1080 Untreated

HT-1080 DMSO

HT-1080 GM6001
