# Supplementary material for: MMP proteolytic activity regulates cancer invasiveness by modulating integrins
Source: Sci Rep. 2017 Oct 27;7:14219. doi: 10.1038/s41598-017-14340-w (PMC5660204; doi:10.1038/s41598-017-14340-w)
Supplement: Supplementary file 1 — Supplementary information [file 41598_2017_14340_MOESM1_ESM.pdf]

Supplementary Text for: MMP proteolytic activity regulates cancer invasiveness by modulating integrins

Alakesh Das, Melissa Monteiro, Amlan Barai, Sandeep Kumar, Shamik Sen*

Department of Biosciences & Bioengineering, IIT Bombay, Mumbai 400076, India

***Correspondence: shamiks@iitb.ac.in**

1. Supplementary Methods

Quantification of focal adhesions: Quantification of number and size of focal adhesions (FAs) was performed using Fiji-Image J software (Supp. Fig. 2). First, background subtraction was performed using the default rolling ball radius of 50 pixels. Next, the processed images (FAs) were thresholded to the same extent. Finally, the number of adhesions per cell was obtained using ImageJ-Analyze Particle tool by choosing a range of 0.25 -10 μm^2 as done elsewhere [1,2] .

2. Supplementary Figures & Figure Legends

Supp. Figure 1

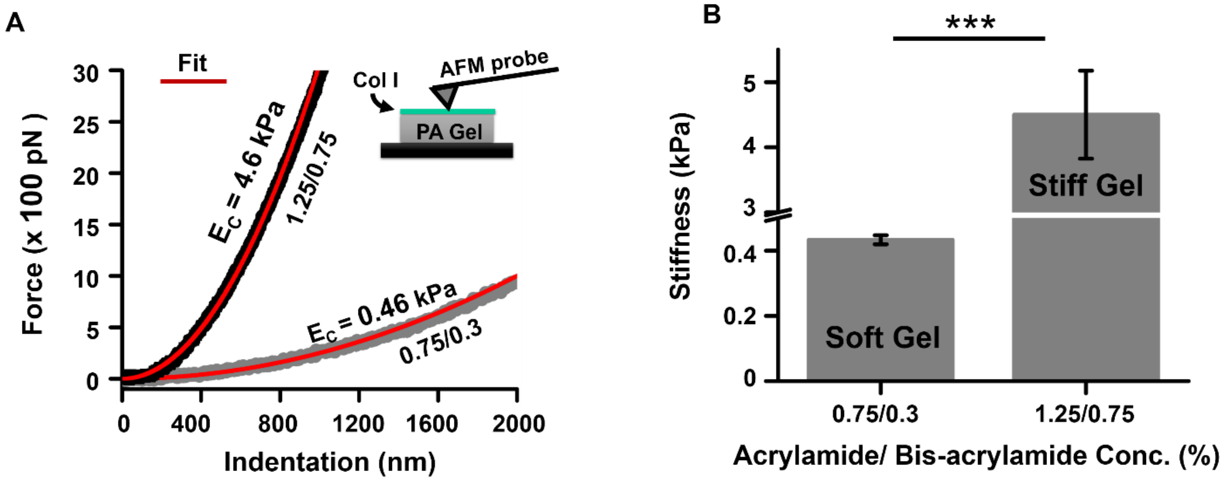

Supp. Figure 1: Stiffness characterization of soft and stiff polyacrylamide (PA) gels. (A) Representative force-indentation curves on soft and stiff PA gel. First 2000 nm of the force curves were fit with Hertz equation to obtain estimates of stiffness. (B) Quantitative analysis of gel stiffness (n = 2, 20-25 indentation points per condition; ***: $p < 0.001$). Statistical significance was determined by one-way ANOVA/Fisher test. Error bars represent standard error of mean (± SEM).

Supp. Figure 2

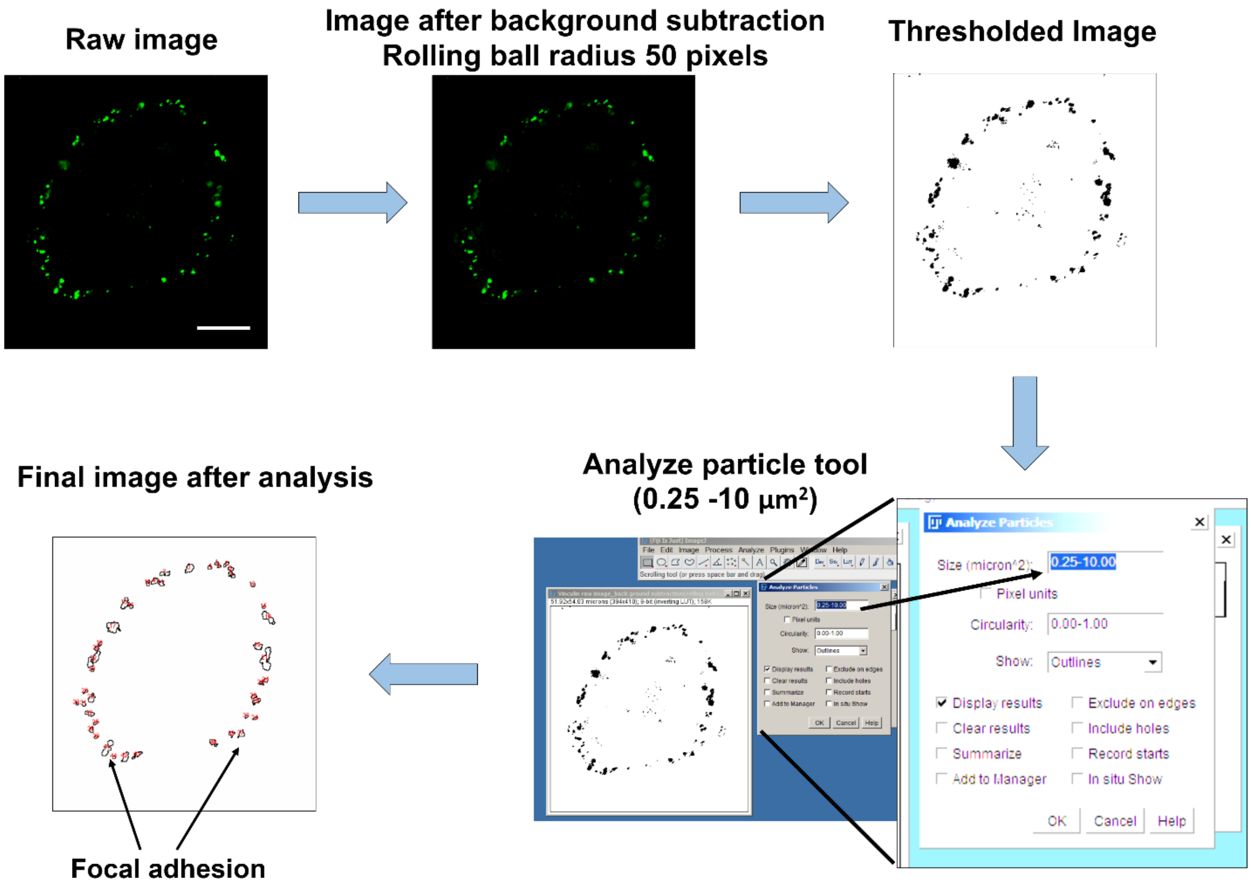

Supp. Figure 2: Steps in quantification of focal adhesions. Raw images (scale bar = 10 µm) are first subjected to background subtraction using the default rolling ball radius of 50 pixels. Next, the processed images (FAs) were thresholded to the same extent. Finally, the number of adhesions per cell was obtained using ImageJ-Analyze Particle tool by choosing a range of 0.25 -10 µm^2**.**

Supp. Figure 3

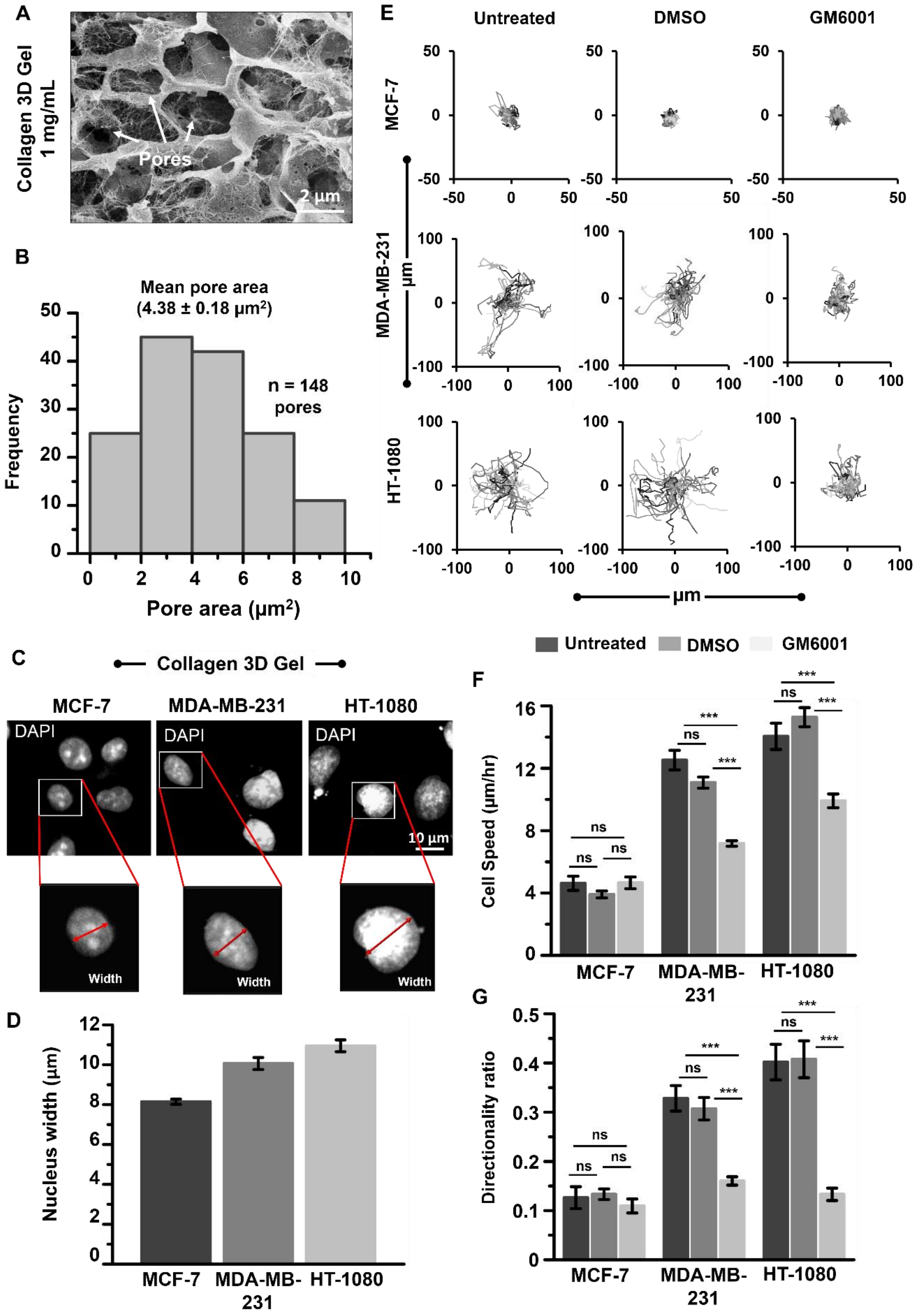

Supp. Figure 3: MMP activity is essential for 3D invasion. (A) Cryo-SEM images of 1 mg/ml collagen gels. (B) Quantification of pore size of 1 mg/ml collagen gels. (C) Representative images of DAPI stained nuclei of MCF-7, MDA-MB-231 and HT-1080 cells embedded in 1 mg/ml collagen gels. Insets show single nuclei with the red lines representing the width of the nucleus (n = 2, 50-70 nuclei per condition). (D) Quantification of nuclear width of MCF-7, MDA-MB-231 and HT-1080 cells. (E) Representative trajectories of untreated, DMSO treated and GM treated cancer cells migrating in 3D collagen gels. (F) Quantification of cell speed of untreated, DMSO treated and GM treated cancer cells (n = 2, 30 cells per condition; ***: $p < 0.001$; **ns**: not significant). Statistical significance was determined by one-way ANOVA/Fisher test. Error bars represent standard error of mean (± SEM). (G) Quantification of migration persistence of untreated, DMSO treated and GM treated cancer cells (n = 2, 30 cells per condition; ***: $p < 0.001$; **ns**: not significant). Statistical significance was determined by one-way ANOVA/Fisher test. Error bars represent standard error of mean (± SEM).

Supp. Figure 4

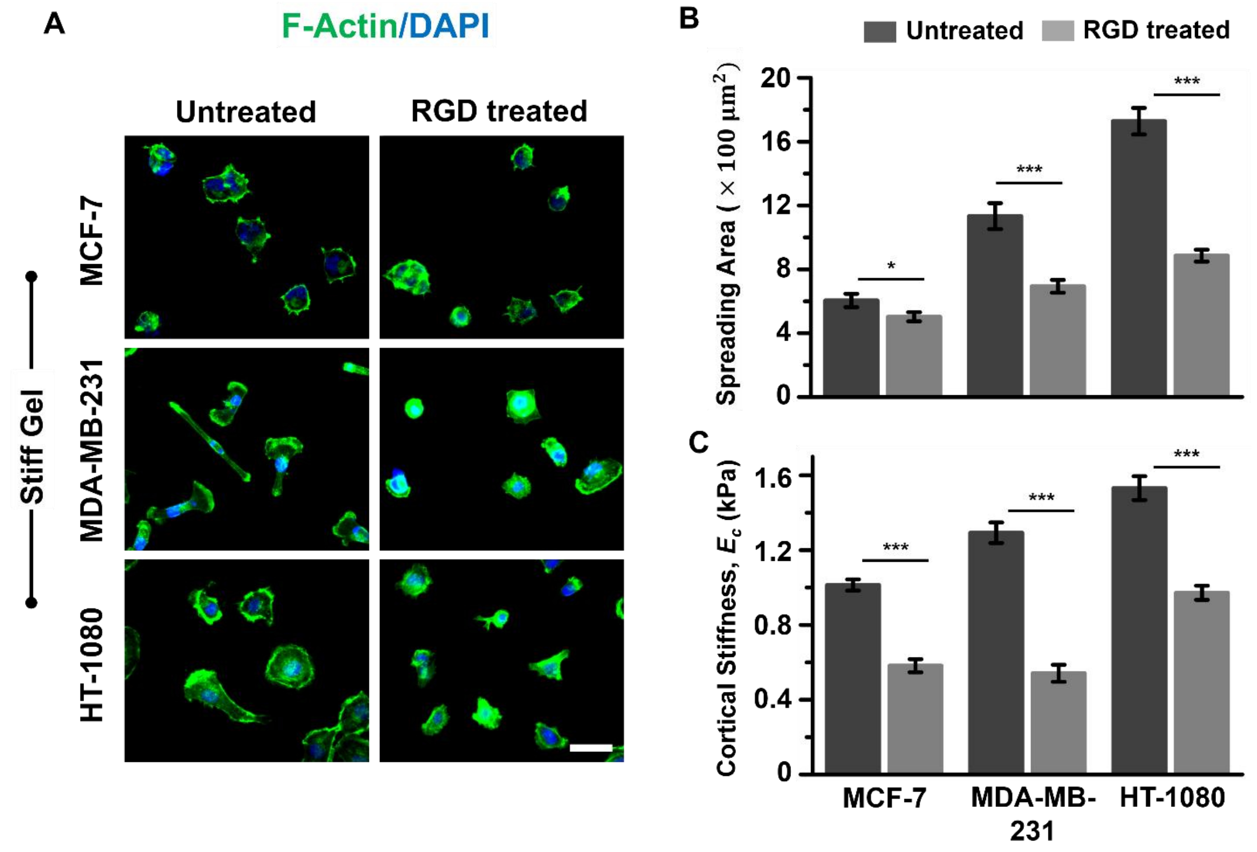

Supp. Figure 4: Integrins are essential for cell spreading and maintenance cell mechanical properties. (A) Representative F-actin (green) and DAPI (blue) stained images of untreated and RGD blocked MCF-7, MDA-MB-231, and HT-1080 cells seeded on stiff gels. Scale bar = 100 µm. (B) Quantitative analysis of untreated and RGD blocked MCF-7, MDA-MB-231 and HT-1080 cancer cells seeded on stiff gels (n = 2-3, at least 130-150 cells per condition). Stars denote statistical significance (***: $p< 0.001$, *: $p< 0.05$). Statistical significance was determined by Mann-Whitney test. Error bars represent standard error of mean (± SEM). (C) Quantitative analysis of cell cortical stiffness of untreated and RGD blocked MCF-7, MDA-MB-231, and HT-1080 cells seeded on stiff gels (n = 3-4, 90-100 cells per condition; ***: $p < 0.001$).Statistical significance was determined by Mann-Whitney test. Error bars represent standard error of mean (± SEM).

Supp. Figure 5

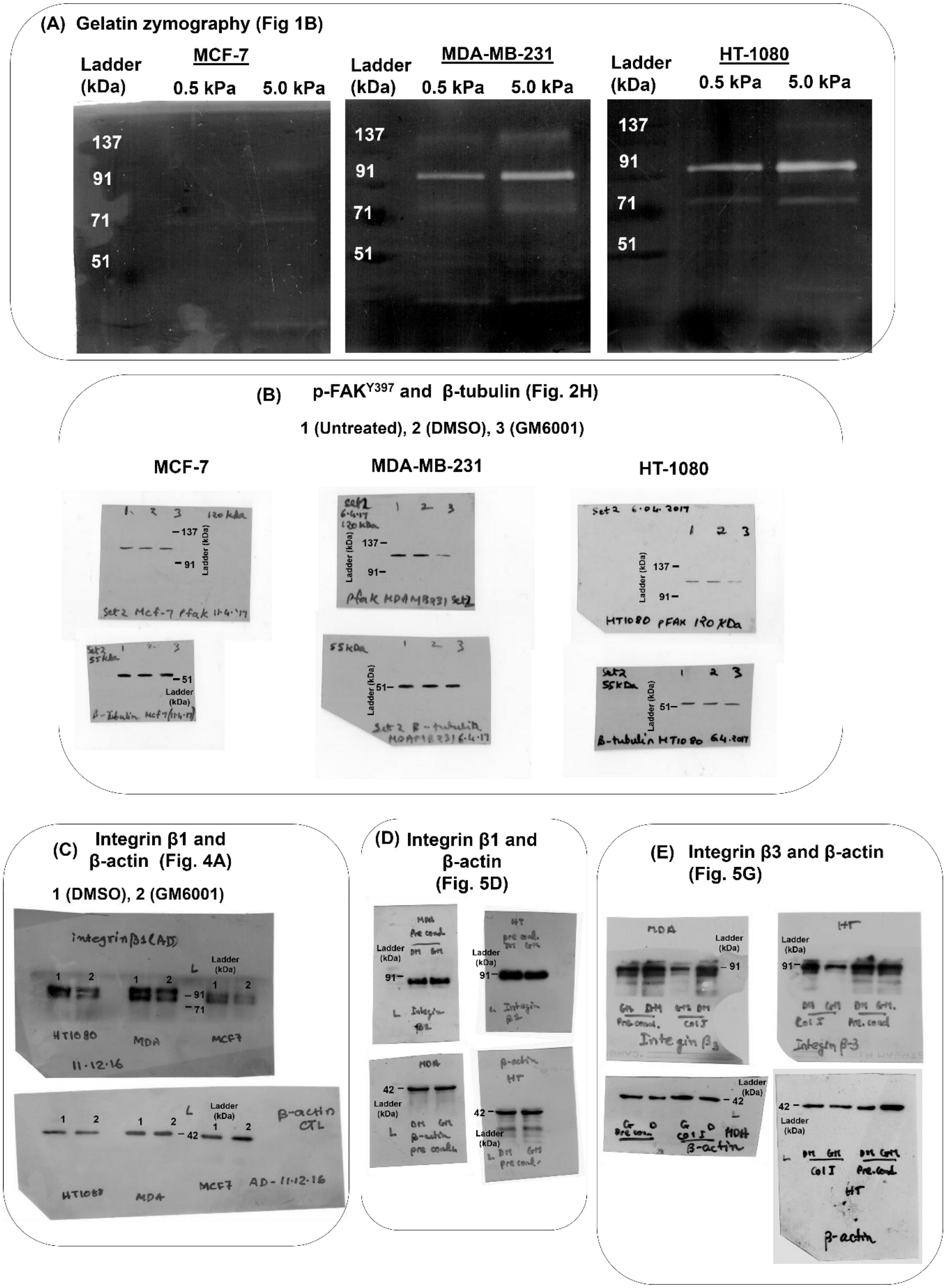

Supp. Figure 5: Orginal scans of blots. (A) Scans of zymograms of MCF-7, MDA-MB-231 and HT-1080 cells. (B) Scans of p-FAK^{Y397} and β-tubulin of untreated, DMSO treated and GM treated cancer cells on col type I stiff gel. (C) Scans of integrin β1 and β-actin of DMSO treated and GM treated cancer cells on col type I stiff gel. (D) Scans of integrin β1 and β-actin of DMSO treated and GM treated cancer cells on pre-conditioned surface. (E) Scans of integrin β3 and β-actin of DMSO treated and GM treated cancer cells on col type I stiff gel and pre-conditioned surface.

3. Supplementary Movie Legends

Supp. Videos V1, V2, V3: Time-lapse movies of untreated, DMSO treated and GM treated MDA-MB-231 cells migrating on stiff gels Multicolored lines represent the migratory tracks.

Supp. Videos V4, V5, V6: Time-lapse movies of untreated, DMSO treated and GM treated HT-1080 cells migrating on stiff gels Multicolored lines represent the migratory tracks.

References

1. Horzum, U., Ozdil, B. & Pesen-Okvur, D. Step-by-step quantitative analysis of focal adhesions. *MethodsX* **1,** 56–59 (2014).

2. Badalà, F., Nouri-mahdavi, K. & Raoof, D. A. Analysis of focal adhesion turnover: A quantitative live cell imaging example. *Methods Cell Biol.* **144,** 724–732 (2008).
